# Exosomal Hsp70 Induces a Pro-Inflammatory Response to Foreign Particles Including Mycobacteria

**DOI:** 10.1371/journal.pone.0010136

**Published:** 2010-04-12

**Authors:** Paras K. Anand, Ellis Anand, Christopher K. E. Bleck, Elsa Anes, Gareth Griffiths

**Affiliations:** 1 Cell Biology and Biophysics Unit, European Molecular Biology Laboratory, Heidelberg, Germany; 2 URIA - Centro de Patogénese Molecular, Faculdade de Farmácia da Universidade de Lisboa e Instituto de Medicina Molecular, Lisbon, Portugal; Institut de Pharmacologie et de Biologie Structurale, France

## Abstract

**Background:**

Exosomes are endosome-derived vesicles that are released when multi-vesicular bodies (MVBs) fuse with the plasma membrane. Exosomes released from mycobacteria-infected cells have recently been shown to be pro-inflammatory. A prominent host molecule that is found within these exosomes is Hsp70, a member of the heat-shock family of proteins.

**Methodology/Principal Findings:**

We first characterized the exosomes purified from control and mycobacteria-infected cells. We found that relative to uninfected cells, macrophages infected with *M. smegmatis* and *M. avium* release more exosomes and the exosomes they released had more Hsp70 on their surface. Both exosomes and exogenous Hsp70 treatment of macrophages led to NF-κB activation and TNFα release in uninfected macrophages; Hsp70 levels were elevated in mycobacteria-infected cells. Macrophage treatment with Hsp70 also led to increase in the phagocytosis and maturation of latex-bead phagosomes. Finally, Hsp70 pre-incubation of *M. smegmatis-* and *M. avium*-infected cells led to increased phago-lysosome fusion, as well as more killing of mycobacteria within macrophages.

**Conclusions/Significance:**

Our results fit into an emerging concept whereby exosomes-containing Hsp70 are effective inducers of inflammation, also in response to mycobacterial infection.

## Introduction

Phagocytosis involves engulfment of particles such as microbes by internalization of a sub-domain of the plasma membrane, resulting in a vesicle called the phagosome. This undergoes a maturation process involving fusion with multiple compartments of the endocytic pathway, of which the last station is the lysosomes [1]. Mycobacteria are prominent examples of bacteria that enter host macrophages by phagocytosis [2], [3]. A striking characteristic of pathogenic mycobacteria is that they inhibit the fusion of their phagosomes with the late endosome/lysosome pathway and, as a consequence, survive and grow within macrophage [1], [4]. One of the mechanisms that contributes to blocking the phagosome-lysosome pathway and subvert the macrophage microbicidal functions is through the lipid-rich cell wall of mycobacteria, which includes components that are immunomodulatory [5], [6], [7]. This lipid-rich cell wall is also prone to shedding within the mildly acidic environment of the early-stage-arrested macrophage phagosome [8]. These components, that include glycopeptidolipids (GPLs), lipoarabinomannan (LAM), phosphatidylinositol mannoside (PIM) and the 19 kDa lipoprotein are carried through into the endosome-phagosome network [8], [9]. From there it has been suggested that they enter MVBs and then are released to the extracellular media within vesicles referred to as exosomes [8], [9], [10].

Exosomes are endosome-derived vesicles (40–100 nm) formed during the formation of multi-vesicular bodies (MVBs). These MVBs can fuse with the plasma membrane, thus releasing their intra-luminal vesicles (ILVs) into the extracellular media, which are then known as exosomes [11], [12]. The importance of these vesicles was first realized when they were isolated from antigen-presenting cells (APCs) and found to be promising in inducing an anti-tumor response [13], [14]. Recently, a few reports have also highlighted the importance of exosomes isolated from macrophages that were infected with mycobacteria. These exosomes were found to be immunomodulatory and could stimulate bystander macrophages and neutrophils to secrete pro-inflammatory mediators, including TNFα and RANTES, and up-regulate iNOS expression [10], [15].

One of the major molecules that are found in these exosomes is macrophage heat shock protein 70 (Hsp70). Members of Hsp family are highly conserved proteins that are found in all prokaryotes and eukaryotes. Hsp70 primarily acts as an intracellular cytoplasmic chaperone and facilitates proper folding of naïve proteins and targets aberrantly folded or damaged proteins for degradation. Although previously considered as mostly an intracellular protein, Hsp70 is up regulated and then released outside cells in exosomes following various kinds of stimuli, notably heat-shock and stress [16], [17], [18]. Studies with exogenous Hsp70 have shown that it can serve to alert the immune system and exert immunomodulatory effects [19], [20], [21]. Recent findings have shown that Hsp70 binds various Toll-like receptors (TLRs) and elicits a rapid intracellular Ca2+ flux, activate NF-κB and thereby up-regulate the expression of pro-inflammatory cytokines and chemokines [22], [23], [24], [25], [26]. Direct and indirect evidence has also implicated the presence of exogenous Hsp70 in promoting phagocytosis of opsonized bacterial particles by mouse macrophages in part by interacting with TLR7 [27], [28].

Since exosomes (that carry Hsp70) from mycobacteria-infected cells were reported to be pro-inflammatory [10], here we confirmed and extended the earlier reports describing the pro-inflammatory nature of exosomes derived from mycobacteria-infected cells. We found that these exosomes stimulated NF-κB activation as well as TNFα release when they were added to control-uninfected macrophages. By immuno-EM and Western-blotting, we observed that infected cells produced more exosomes as compared to exosomes from control-uninfected cells and these exosomes had Hsp70 on their surface. Further, we observed a selective activation by purified Hsp70 of a part of the pro-inflammatory response in macrophages infected with mycobacteria; there, Hsp70 induced activation of NF-κB as well as TNFα, but not IL-1β. Further, exogenous Hsp70 treatment led to increased phagocytosis as well maturation of latex-bead phagosomes by RAW 264.7 mouse macrophages. Similarly, this treatment led to increased maturation of *M. smegmatis* and *M. avium* phagosomes as well as more killing of the bacteria. These data add to an emerging view of exosomes and their major component Hsp70 as being effective inducers of the inflammatory response, and demonstrate that phagosome-lysosome fusion is an integral part of this response.

## Results

### Exosomes derived from mycobacteria-infected cells are pro-inflammatory

**Figure 1:**
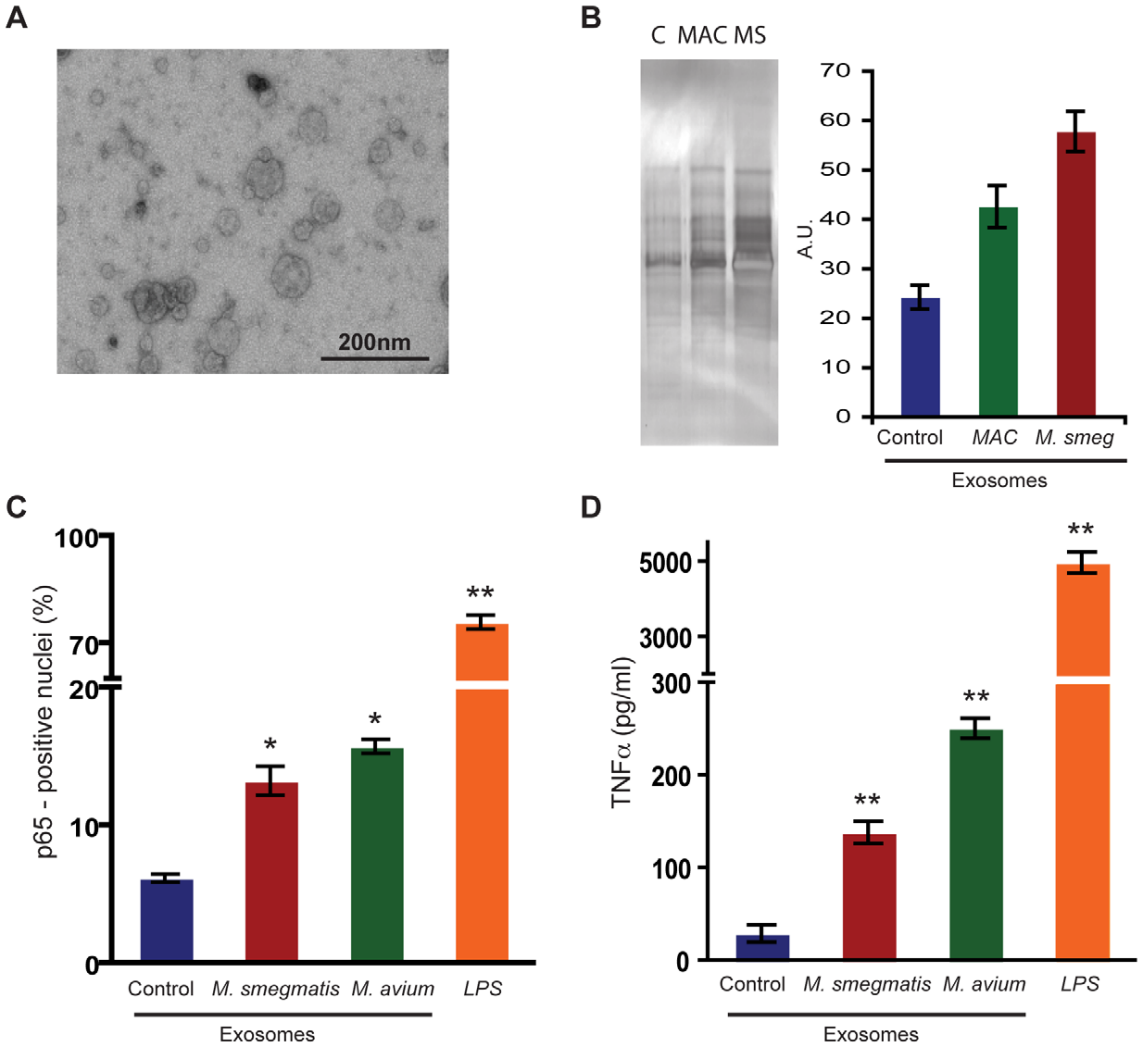
Exosomes derived from mycobacteria-infected cells are pro-inflammatory. **A.** RAW264.7 mouse macrophages were cultured in DMEM media depleted of endogenous exosomes in the serum. After 48 h, exosomes were purified by differential centrifugation. The EM micrograph shows a representative image of negatively-stained exosomes. **B.** Cells were either left uninfected or infected with *M. smegmatis* (MS) or *M. avium* (MAC). After 48 h of infection, exosomes were isolated and total exosomes were subjected to SDS-PAGE followed by silver staining. Histogram (right panel) shows the quantitative analysis of the total densities of all protein lanes, as measured using Image J program. **C.** RAW 264.7 cells were incubated with exosomes isolated from control, *M. smegmatis* and *M. avium*–infected macrophages or subjected to LPS (100 ng/ml) treatment. After 24 h, cells were immunolabeled with anti-p65 antibody. Data show percentage of cells showing p65-positive nuclei (± SEM) from experiments done three independent times. At least 100 cells were counted per condition. * p≤0.05, ** p≤0.01. **D.** RAW264.7 cells were incubated with exosomes isolated from control, *M. smegmatis* and *M. avium*–infected macrophages or subjected to LPS (100 ng/ml) treatment. After 24 h, supernatants were collected and assayed for TNFα activity using ELISA. Data represent mean ± SEM of three independent experiments. * p≤0.05, ** p≤0.01.

We first isolated exosomes from control uninfected and mycobacteria -infected (*M. smegmatis* and *M. avium*) RAW 264.7 mouse macrophages. Negative stain EM analysis confirmed that the fraction consisted of vesicles that were mostly in the 40–100 nm in size (**Fig. 1A**). Uninfected cells did not secrete many exosomes. After infection with *M. smegmatis* as well as *M. avium* at MOI of 10∶1, the amount of exosomes released by both types of infected-macrophages increased approximately three-to-four times relative to control cells, as evaluated by micro-BCA assay of the total exosome content (**Table 1**). This increase in exosome release was not dependent on number of bacteria within cells as infection with *M. smegmatis* at different MOIs in the range of 1∶1 to 20∶1 gave similar amounts of exosome release (results not shown). When we silver-stained the gel loaded with a representative exosome preparation, we observed a similar increase in exosome proteins from infected macrophages relative to controls (**Fig. 1B**). The histogram in **Fig. 1B** shows the densitometry scanning of the total densities of all the bands in the exosome gels using the Image J program.

**Table 1:** Amount of exosomes obtained from different conditions.

Condition	Approximate cell number/Volume of DMEM media	Concentration (micro-BCA assay)
Control	2×10^8^/25 ml	8±2 µg/ml
Latex-Beads	2×10^8^/25 ml	19±2 µg/ml
*M. smegmatis*	2×10^8^/25 ml	34±4 µg/ml
*M. avium*	2×10^8^/25 ml	23±3 µg/ml
LPS	2×10^8^/25 ml	39±4 µg/ml

Concentration of exosomes (as measured by micro-BCA assay) isolated after 48 h from control, latex-bead incubated, *M. smegmatis* and *M. avium* infected or LPS treated mouse macrophages.

Next, we asked whether these three different types of exosomes could initiate a pro-inflammatory response. For this, we first tested the pro-inflammatory transcription factor NF-κB. NF-κB family is made of five subunits: p50, p52, p65 (RelA), RelB, and c-Rel. However, only p65, RelB and c-Rel possess the trans-activation domain and thus act as potent transcriptional activators by themselves [29]. Most commonly, the NF-κB transcription factor is present in the cytoplasm as heterodimers of p65 and p50 subunits in a complex with the inhibitor IκB. Upon activation through cell surface receptors such as TLRs, these dimers translocate into the cell nucleus and transcribe many genes involved in generating a pro-inflammatory response [30].

We first used indirect immunofluorescence microscopy to quantitate the number of cells that show p65 translocation from the cytoplasm into the nucleus in response to the three different types of exosomes; the fraction of cells with p65 in the nucleus is a sensitive measure of NF-κB activation [31]. For this, the macrophage cultures were incubated with 10 µg of each kind of exosome. After 24 h of incubation, the cells were immunolabeled with anti-p65 antibody. Cells that were incubated with exosomes from *M. smegmatis* and *M. avium* infected macrophages showed a higher percentage of p65 translocation into the nucleus of untreated cells than to those cells that were incubated with exosomes isolated from control cells (**Fig. 1C**). The classical inducer of NF-κB, bacterial LPS, gave a strong activation, as expected (**Fig. 1C**).

Activation of NF-κB leads to induction of many hundred genes including lysosomal enzymes, membrane-trafficking proteins and cytokines [31]. Increased synthesis of these proteins enhances phagosome maturation, that helps macrophages to kill non-pathogen *M. smegmatis* [31]. However, pathogenic mycobacteria such as *M. tuberculosis* or *M. avium* inhibit NF-κB activation and therefore likely survive in an environment generating little or no pro-inflammatory response [31], [32]. A prominent down-stream target of NF-κB activation is the synthesis of the cytokine TNFα that is secreted by the macrophage and is a major part of pro-inflammatory response of NF-κB. We therefore measured TNFα in the supernatants from the different sets of macrophages that were treated with the different sources of exosomes, using ELISA. We observed that cells that were incubated with exosomes released by *M. smegmatis* or *M. avium*–infected cells secreted significantly more TNFα in the supernatant, relative to cells incubated with exosomes from control cells (**Fig. 1D**). The amount of TNFα seen with *M. avium*- or *M. smegmatis*- cell exosomes was still only a small fraction of the value seen with LPS (**Fig. 1D**). Thus, although the pathogen *M. avium* inhibits the NF-κB pro-inflammatory response more than *M. smegmatis* [31], *M. avium*-infected cells are able to release appreciably more (pro-inflammatory) exosomes than those infected with *M. smegmatis* and these exosomes stimulate more TNFα.

Given that mycobacterial lipids are released from the surface of the bacteria within phagosomes [8], [9] we asked whether these lipids were found in appreciable amounts in the exosomes we isolated from mycobacteria-infected macrophages. For this we tested the reactivity of the different types of exosomes against an anti-Ara-LAM antibody. This antibody recognizes both Ara-LAM as well as Man-LAM by Western blotting (results not shown). We observed that there was no signal for LAM observed in the exosomes (10 µg) from uninfected (control) cells or in those from *M. smegmatis*- infected cells. A faint band, corresponding to significantly less than 20 ng was detected in exosomes from *M. avium*–infected cells (**Fig. S1**). From these data we conclude that in the *M. smegmatis* exosomes LAM is unlikely to be important in the pro-inflammatory response; however, we cannot rule out that the low levels detected in the *M. avium* phagosomes may contribute to some of the inflammatory effects we measured.

In parallel, we also tested whether phagocytosis of inert particles, latex beads, could induce exosome release from macrophages. For this, we incubated the cells with avidin-conjugated latex-beads. After 1 h of phagocytosis, the medium was replaced and then finally collected for exosome isolation after 48 h. We found that even phagocytosis of beads could lead to induction of exosome release at a level significantly higher than that released from control-uninfected macrophages. We also tested LPS in these experiments and found that the amount of exosomes released was the highest of all the conditions tested (**Table 1**).

### Hsp70 is present on exosomes

**Figure 2:**
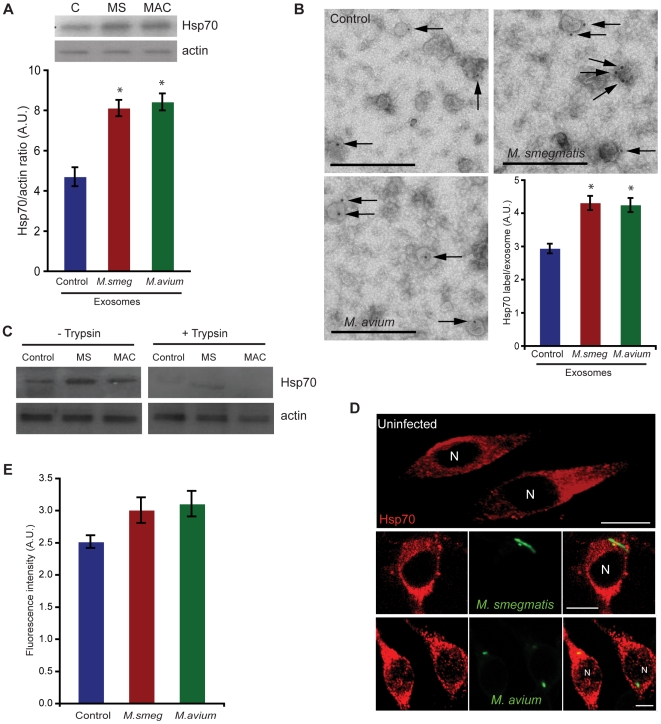
Hsp70 is present on exosomes. **A.** Cells were either left uninfected (C; control) or infected with *M. smegmatis* (MS) or *M. avium* (MAC). After 48 h of infection, exosomes were isolated and total exosomes were Western-blotted with anti-Hsp70 and anti-actin antibody. Upper panel; Representative blot from experiment done three times. Lower panel; Densitometric quantitation of the individual Hsp70 protein bands (relative to actin) as measured by Image J. **B.** Exosomes isolated from control macrophages or *M. smegmatis* or *M. avium* – infected macrophages were surface - immunolabeled with anti-Hsp70 antibody and visualized by transmission electron microscopy. Representative images from each of the three different conditions. Lower right panel, Data represent Hsp70 (gold) labeling density, given as gold per average vesicle on the surface of each kind of exosome. Scale bars–500 nm. **C.** Exosomes from three different conditions were subjected to trypsin proteolysis for 5 min at 37°C. 10 µg of exosomes (before and after trypsin) from each condition was subjected to SDS-PAGE and immunoblotted with anti-Hsp70 and anti-actin antibody. **D.** Cells were either uninfected or infected with GFP - *M. smegmatis* or rhodamine - *M. avium*. After 24 h, the cells were labeled with anti-Hsp70 antibody for total fluorescence. Note that rhodamine–*M. avium* (green) and Hsp70 labeling (red) is shown in false color. Images show representative confocal images. N, nucleus. Scale bars–10 µm. **E.** Cells grown in 24-well tissue culture plates were infected and immunolabeled as above in 2D. The total fluorescent intensity was measured by Tecan plate reader (Safire 2). Data represent mean ± SEM of three independent experiments.

Many reports in the literature indicate that the major component of exosomes is Hsp70 and that this chaperone can activate many signaling pathway [16], [17], [18], [20], [22], [23], [33]. In order to investigate the presence of Hsp70 in exosomes, we subjected the three different types of exosomes to immuno-blotting with anti-Hsp70 antibody (**Fig. 2A**). This experiment revealed that Hsp70 is present on all three types of exosomes. However, we observed that approximately two-times more Hsp70 is released from infected macrophages than control macrophages (**Quantitation, lower part of** **Fig. 2A**). Similarly, we also observed higher Hsp70 expression in exosomes from latex-bead incubated cells than control cells (**Fig. S2**). Exosomes from LPS-treated cells also showed an elevated Hsp70 expression (**Fig. S2**).

Next, we carried out immuno-electron microscopy on these exosome vesicles isolated from control and infected cells and adsorbed onto EM grids. In this method only Hsp70 exposed on the outside of exosomes is accessible for labeling. Some surface Hsp70 could be detected on all types of exosomes (**Fig. 2B**). However, Hsp70 was present mainly on the surface of exosomes isolated from infected macrophages. A quantitative approach to measure the gold-label on exosomes revealed higher surface labeling of Hsp70 on exosomes from *M. smegmatis* or *M. avium – *infected cells than exosomes from control cells (**Fig. 2B** **, lower right panel**).

Hsp70 can be both a soluble as well as membrane-bound protein [34], [35]. In order to show the surface-presence of Hsp70 conclusively, we subjected the three different kinds of exosomes to a mild-trypsin treatment. We hypothesized that the exosomal surface-Hsp70 will be sensitive to trypsin proteolysis. Indeed exposure of exosomes to 0.25% trypsin resulted in significant reduction in the levels of exosomal Hsp70 as seen by immunoblotting (**Fig. 2C**).

Next, we immunolabeled macrophages with anti-Hsp70 followed by fluorescently- tagged secondary antibody (**Fig. 2D**). By this approach, we compared the total fluorescence of uninfected cells with cells infected with *M. smegmatis* or *M. avium* using a plate-reader. The *M. smegmatis* or *M. avium*–infected cells showed marginally higher Hsp70 total fluorescence than control uninfected cells (**Fig. 2E**).

**Figure 3:**
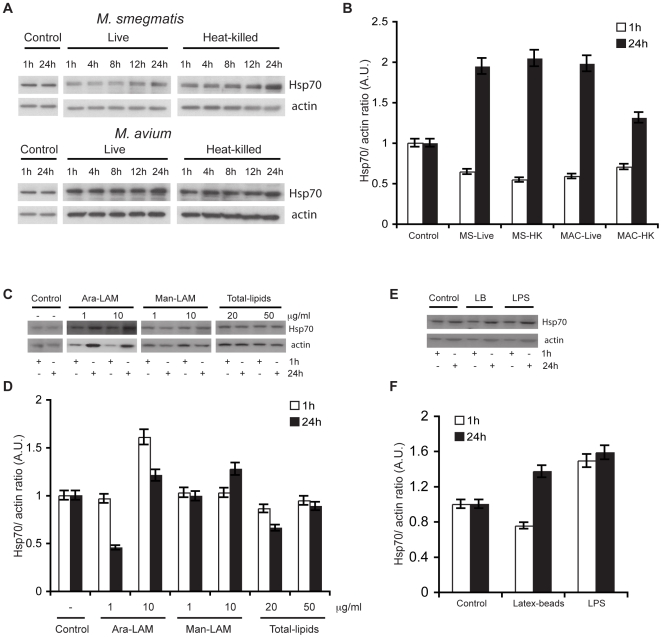
Infection with mycobacteria up-regulates Hsp70 expression. **A.** RAW 264.7 cells were either uninfected (control) or infected with live or heat-killed (HK) *M. smegmatis* or *M. avium*. At different times after infection, cell lysates were prepared. The protein preparations were loaded on SDS-PAGE and immunoblotted with anti-Hsp70 and anti-actin antibody. A representative blot from two-independent experiments is shown. **B.** The above blot was densitometrically scanned by Image J. Data represent mean (± SEM) ratio of Hsp70 to the loading control actin in each sample. **C.** RAW cells were either left untreated (control) or treated with Ara-LAM (1 or 10 µg/ml), Man-LAM (1 or 10 µg/ml) and total mycobacterial lipids (20 or 50 µg/ml). At 1 h and 24 h after infection, cell lysates were prepared that were subjected to SDS-PAGE and immunoblotted with anti-Hsp70 and anti-actin antibody. A representative blot from two independent experiments is shown. **D.** The above blot was densitometrically scanned by Image J. Data represent mean (± SEM) ratio of Hsp70 to the loading control actin in each sample. **E.** RAW cells were either left untreated (control) or incubated with latex-beads or incubated with LPS (100 ng/ml). At 1 h and 24 h, cell lysates were prepared and loaded onto SDS-PAGE. Immunoblotting of the membranes was performed with anti-Hsp70 and anti-actin antibody. A representative blot from two independent experiments is shown. **F.** The above blot was densitometrically scanned by Image J. Data represent mean (± SEM) ratio of Hsp70 to the loading control actin in each sample.

In order to further evaluate the intracellular levels of Hsp70 expression, we made protein preparations of control, *M. smegmatis* and *M. avium* infected macrophages at different times after infection and blotted them with anti-Hsp70 antibody (**Fig. 3A**). Although not much change was found at earlier times after infection, densitometric quantitation of the protein-bands revealed that there was a significant increase in total Hsp70 (relative to total actin levels) present within cells after 24 h of infection with *M. smegmatis* or *M. avium* (**Fig. 3B**). These data argue that more Hsp70 is synthesized in infected cells, and more of this is then released extracellularly via exosomes. Further, as shown in **Fig. 3B** this increase in expression was independent of whether the bacteria were live or dead (**Fig. 3B**). This suggests that the binding of the mycobacteria to the cells is sufficient to stimulate the up-regulation of the heat shock protein (**Fig. 3B**).

Since a small amount of LAM could be detected in the exosomes isolated from *M. avium*- infected cells, we next tested if mycobacterial lipids such as LAM or total mycobacterial lipids could also induce Hsp70 expression. For this, the macrophages were incubated with either Ara-LAM or Man-LAM or total- mycobacterial- lipids at different concentrations (**Fig. 3C, D**). After 1 h and 24 h of incubation, the total cell lysates were subjected to immunoblotting with anti-Hsp70 antibody. At a concentration of 1 µg/ml, there was not much change in Hsp70 expression after 1 h treatment with Ara-LAM. However, the levels of Hsp70 expression decreased after 24 h. At 10 µg/ml of Ara-LAM, the protein expression increased significantly at 1 h. There was not much change in Hsp70 levels after incubation with Man-LAM, with either, 1 or, 10 µg/ml. When macrophages were exposed to total-lipids the effects were minimal with both 20 and 50 µg/ml (**Fig. 3C, D**). Overall, these data argue that any mycobacterial glycolipids exposed on the outside of exosomes would be expected to have little capacity to stimulate an increase in Hsp70 levels. In contrast to these lipids both LPS at 1 h and 24 h and latex beads at 24 h, but not 1 h, could significantly increase the levels of this heat shock protein (**Fig. 3E, F**).

### Pro-inflammatory effects of Hsp70

**Figure 4:**
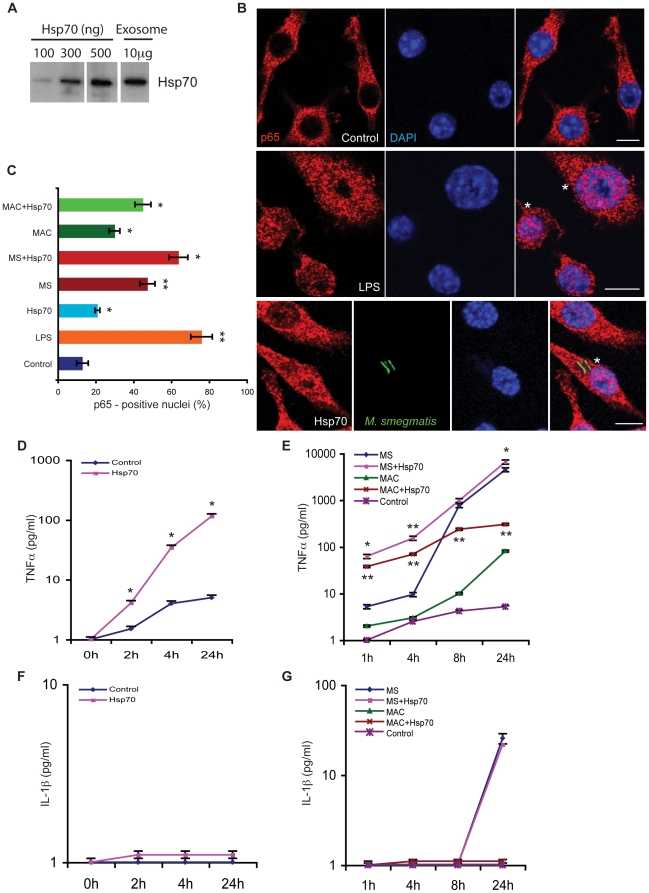
Pro-inflammatory effect of Hsp70 on control and infected cells. **A.** Representative immunoblot showing reactivity of purified Hsp70 and 10 µg exosomes with anti-Hsp70 antibody on SDS-PAGE. **B.** RAW 264.7 macrophages were untreated (control) or treated with LPS (100 ng/ml for 2 h) or with Hsp70 (100 ng/ml for 2 h). Similarly, cells pre-treated or not with Hsp70 were infected either with *M. smegmatis* or *M. avium*. After 1 h of infection, cells were immunolabeled with anti-p65 antibody (red). This image shows representative confocal images showing p65- labeling in control, LPS and Hsp70-treated *M. smegmatis*-infected macrophages. Asterisks show cells with p65 in the nucleus. Scale bars–10 µm. **C.** Quantitative analysis of the p65- positive nuclei in different conditions is shown. Data represent mean ± SEM from three different experiments. * p≤0.05, ** p≤0.01. **D.** Untreated cells (control) or cells treated with Hsp70 were left uninfected or (**E**) infected with *M. smegmatis* or *M. avium* (MAC). At the indicated times, supernatants were collected and assayed for TNFα using ELISA. Data represent mean ± SEM from three different experiments. * p≤0.05, ** p≤0.01. **F, G.** As in 4D,E; the supernatants were collected and assayed for IL-1β using ELISA.

Hsp70 is known to initiate signaling pathways downstream of TLR2 and TLR4 [36], [37]. To investigate if it also leads to generation of pro-inflammatory response in our system, we used commercially available mouse Hsp70 to treat macrophages. First, we titrated purified Hsp70 on a protein gel and then immuno-blotted it along with 10 µg of exosomes against anti-Hsp70 antibody (**Fig. 4A**). We observed that approximately 500 ng of Hsp70 corresponds to the levels found within 10 µg exosomes. For the experiments carried out below we used 100 ng Hsp70, which was the minimum level we could detect with the anti-Hsp70 antibody and that also gave the stimulatory effects described below.

We next incubated macrophages with exogenous Hsp70 and then measured NF-κB activation in these cells after 2 h using anti-p65 antibody followed by labeling with fluorescently tagged secondary antibody. As a positive control we tested LPS, a known potent inducer of NF-κB (**Fig. 4B**). The percentage of macrophages that showed NF-κB activation after 2 h was significantly higher in cells treated with Hsp70, relative to control-uninfected macrophages (∼22% of cells showed nuclear labeling vs. 13% in the control) (**Fig. 4C**). A more significant increase in nuclear p65 was seen in *M. avium*- infected cells (30%) and this level rose to 48% (**Fig. 4C**) when the macrophages were infected with *M. smegmatis,* as expected [31]. The addition of Hsp70 to *M. avium*- infected cells enhanced NF-κB nuclear activity that was more striking with *M. smegmatis*- infected cells, where the level of nuclear NF-κB was almost at the same level as seen with LPS stimulation (**Fig. 4C**). Thus, Hsp70 can synergize with mycobacterial infection to enhance the inflammatory response.

Uninfected macrophages that were incubated with Hsp70 also released more TNFα in the supernatant than control cells (**Fig. 4D**). It is known that infection with *M. smegmatis* induces a potent synthesis and release of TNFα activity, whereas *M. avium* is a poor inducer [32]. As shown in **Fig. 4E**, TNFα release was also significantly higher in cells that were infected with *M. smegmatis* in the presence of Hsp70. A significant, but not quantitatively striking effect on TNFα release was seen in *M. avium* infected macrophages that were treated with Hsp70 (**Fig. 4E**).

We also tested the release of another cytokine IL-1β under these conditions. Of all the conditions tested, only cells that were pre-incubated with Hsp70 and infected with *M. smegmatis* showed small but detectable IL-1β release at 24 h (**Fig. 4F, G**) Pro-IL-1β is not expressed constitutively and transcriptional induction is required, most commonly via TLRs. When we tested IL-1β release with Hsp70 treatment in LPS primed macrophages, we did not see any significant increase in IL-1β release relative to control cells (**Fig. S3**). Another step of regulation for this cytokine is at the post-translational level where effectors such as extracellular ATP activate caspase-1 that cleaves pro- IL-1β to its mature form, thereby releasing it into the medium. Our data also argue that Hsp70 is neither able to transcribe IL-1β mRNA synthesis nor induce the caspase-1-mediated proteolysis of pro-IL-1β.

### Exogenous Hsp70 leads to increase in phagocytosis and maturation of latex-bead phagosomes (LBPs)

**Figure 5:**
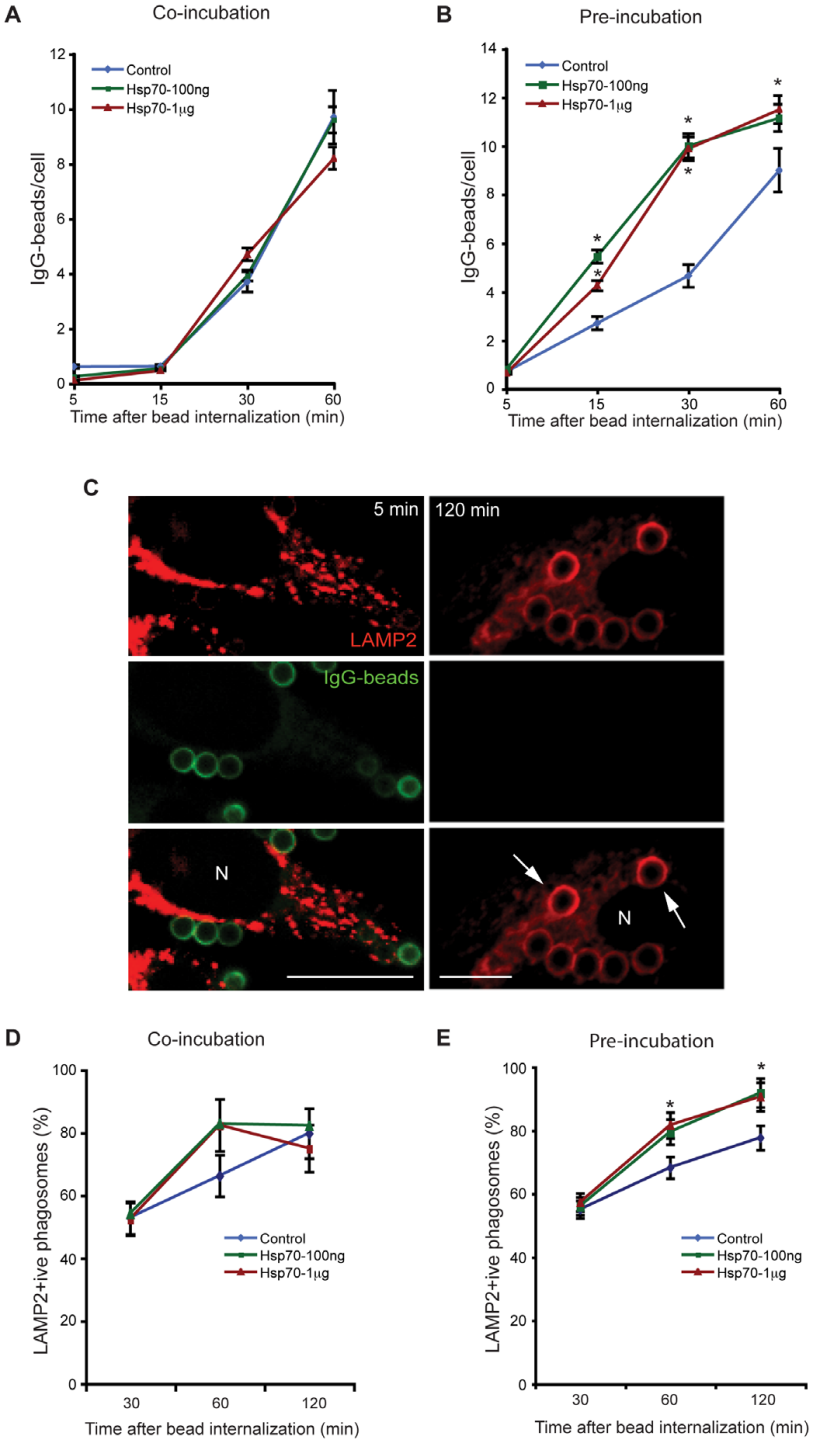
Hsp70 promotes increased phagocytosis of latex-beads and maturation of latex-bead phagosomes (LBP). **A.** Cells were either co-incubated or, **B.** pre-treated for 2 h with Hsp70 before feeding them with IgG-coated latex-beads. At different times after bead internalization, cells were fixed with 3.7% paraformaldehyde. The outside beads were differentially labeled with Alexa Fluor-488 conjugated anti-IgG antibody without permeabilizing the cells. Data represent mean ± SEM from two independent experiments. At least 20 fields per coverslip were analyzed. * p≤0.05. **C.** After labeling the outside beads with alexa-Fluor 488 conjugated anti-IgG antibody, the cells were permeabilized with 0.1% saponin followed by immunolabeling with anti-LAMP2 antibody. The figure shows confocal microscope images taken after 5 min and 120 min of bead uptake. Arrows show fully matured LAMP2 – positive latex-bead phagosomes. Scale bars–10 µm. **D.** Quantitative kinetic analysis of LAMP2–positive phagosomes in Hsp70 co-incubated, or **E.** Hsp70 pre-treated macrophages. Data represent mean ± SEM from two different experiments. * p≤0.05.

Since phagocytosis has been shown to be a part of the pro-inflammatory process [38], we next asked whether Hsp70 has any effect on this process in our system. First, we tested IgG-coated latex beads. We used two different approaches: 1) Macrophages were co-incubated with exogenous Hsp70 and latex-beads simultaneously, and 2) Macrophages were pre-incubated with Hsp70 for 2 h before feeding them with latex beads. At different times after bead-uptake, cells were fixed and the number of beads phagocytosed was quantitated as described in detail in experimental procedures. Whereas co-incubation of Hsp70 and latex beads simultaneously with macrophages did not result in increase of latex-bead uptake (**Fig. 5A**), we found that significant increase in uptake occurred after pre-incubation of Hsp70 (**Fig. 5B**).

To evaluate if this increased phagocytosis also translates into increased maturation of the LBPs, cells were stained with anti-LAMP2 antibody at different times after bead internalization (**Fig. 5C**). LAMP2 is a late- endosome-lysosome marker and its presence on phagosomes is an indication that phagosomes have fused with lysosomes. Quantitation of LAMP2 - positive phagosomes revealed that a significantly higher percentage of phagosomes had matured in cells that were pre-incubated with Hsp70 at 120 min of bead uptake (**Fig. 5D, E**).

### Exogenous Hsp70 leads to increased phago-lysosome fusion of mycobacterial phagosomes

**Figure 6:**
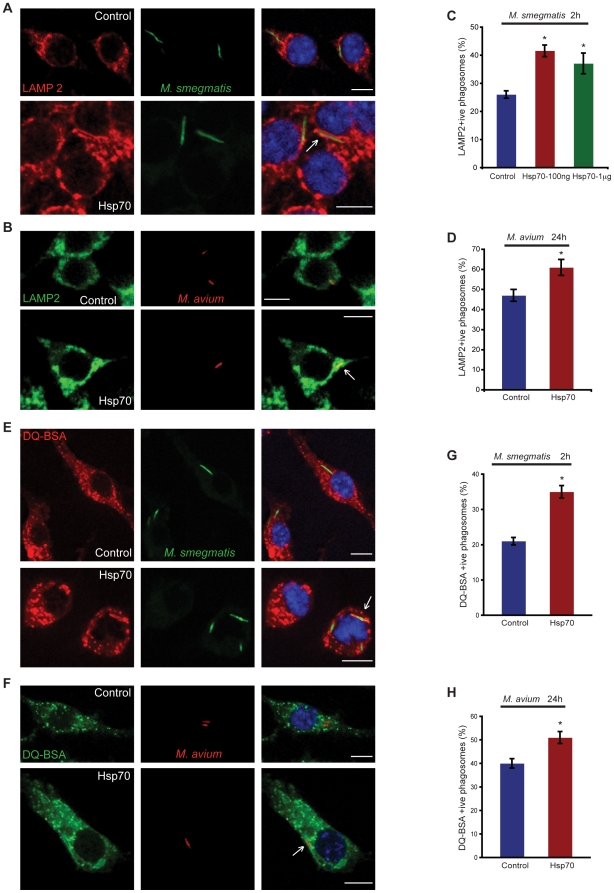
Hsp70 promotes maturation of M. smegmatis and M. avium phagosomes. **A.** Macrophages grown on coverslips were pre-treated or not with Hsp70. After 2 h, cells were infected with GFP - *M. smegmatis* or **B.** rhodamine- *M. avium.* At 2 h (*M. smegmatis*) or 24 h (*M. avium*) after infection, cells were subjected to indirect immunofluorescence microscopy against LAMP2. Arrow shows fully matured LAMP2 – positive mycobacteria phagosomes. Scale bars–10 µm. **C.** Quantitative analysis of LAMP2–positive GFP- *M. smegmatis* or **D.** rhodamine- *M. avium* phagosomes after 2 h or 24 h of infection, respectively. Data represent mean ± SEM from three independent experiments. At least 100 phagosomes were counted per condition. * p≤0.05. **E.** As in Fig. 6A; macrophages were further pre-incubated with 10 µg/ml DQ-BSA 2 h before *M. smegmatis* or **F.** *M. avium* infection. Arrow shows fully matured DQ-BSA – positive *M. smegmatis* or *M. avium* phagosomes. Scale bars–10 µm. **G.** Quantitative analysis of DQ-BSA –positive *M. smegmatis* or **H.** *M. avium* phagosomes after 2 h or 24 h of infection. Data represent mean ± SEM from three independent experiments. At least 100 phagosomes were counted per condition. * p≤0.05.

Due to its appreciable effect on phagocytosis and maturation of LB, we next investigated whether exogenous Hsp70 can also lead to increased uptake of mycobacteria and maturation of mycobacterial phagosomes. To test this, we first infected macrophages, that were pre-treated with Hsp70 for 2 h, with GFP- *M. smegmatis* (**Fig. 6A**) or rhodamine-labeled–*M. avium* (**Fig. 6B**). After 2h (*M. smegmatis*) or 24 h (*M. avium*), the cells were fixed and immunolabeled with anti-LAMP2 antibody (**Fig. 6A,B**). Quantitation of LAMP2- positive *M. smegmatis* or *M. avium* phagosomes revealed that a significant percent of phagosomes (*M. smegmatis* >40%; *M. avium* >60%) were now matured in macrophages that were incubated with Hsp70 compared to control cells (**Fig. 6C,D**). A higher concentration of Hsp70 did not lead to further increase in maturation of *M. smegmatis* phagosomes (**Fig. 6C**).

In another experiment done exactly as above, we also incubated the macrophages with 10 µg/ml of fluorogenic protease substrate DQ-BSA 2 h before infection (**Fig. 6E,F**). This, initially non-fluorescent compound enters lysosomes after fluid-phase endocytosis and when exposed to protease activity it becomes cleaved and thereby fluorescent. Quantitation of DQ-BSA-positive phagosomes revealed that significantly higher percent of phagosomes (*M. smegmatis* >35%; *M. avium* >50%) acquired protease activity and were therefore fused with lysosomes in Hsp70-treated macrophages as compared to control cells (**Fig. 6G,H**). These results agree well with those seen with LAMP 2 labeling and argue that Hsp70 binding to macrophages enhances phago-lysosome fusion.

### Exogenous Hsp70 leads to increased killing of mycobacteria

**Figure 7:**
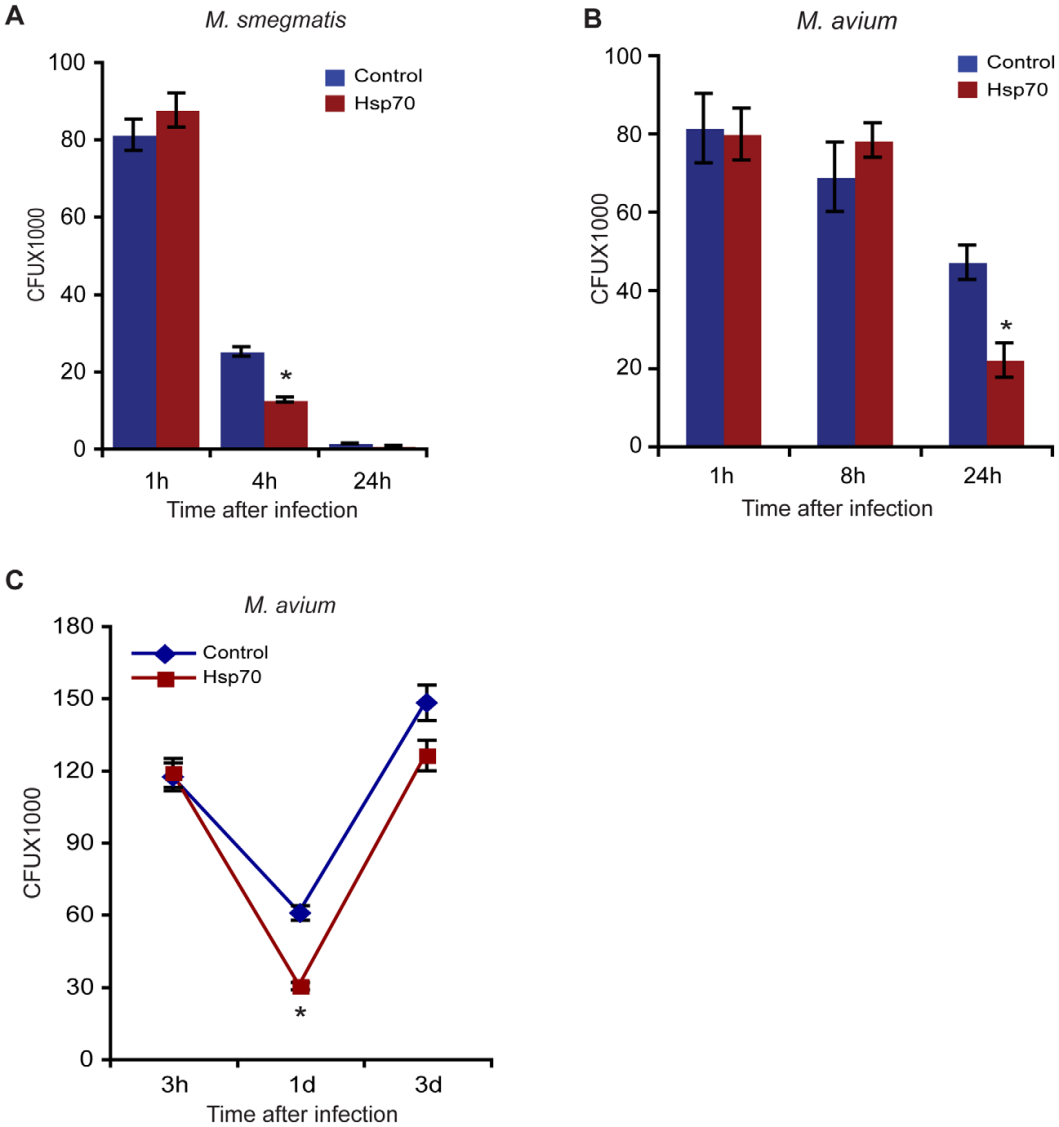
Hsp70 promotes mycobacterial killing. **A.** Macrophages were pre-treated or not with Hsp70 followed by infection with GFP- *M. smegmatis*. At indicated times after infection, CFU were determined. Data represent mean ± SEM from three independent experiments. *p≤0.05. **B.** Macrophages were pre-treated or not with Hsp70 followed by infection with *M. avium*. At the indicated times (1 h, 8 h and 24 h), or **C.** (3 h, 1 d and 3 d) after infection, CFU were determined. Data represent mean ± SEM from three independent experiments. *p≤0.05.

An increase of phago-lysosome fusion in mycobacteria-infected cells is usually correlated with more killing of mycobacteria [39], [40]. We therefore next asked whether Hsp70 has any effect on bacterial killing, using colony -forming units (CFU) as a read-out. For this, we again used *M. smegmatis*, a non-pathogenic bacteria that is killed within macrophages and *M. avium*, a pathogenic mycobacteria that tends to resist fusion with lysosomes. Within macrophages, *M. smegmatis* is killed naturally by 24 h [40]. However, *M. avium* shows an initial killing at 1 day after infection followed by increased growth at 3 days of infection [41]. In these experiments, pre-treatment with Hsp70 did not significantly affect the rate of uptake of *M. smegmatis* and *M. avium* by macrophages, as is evident at the 1 h infection time-point (**Fig. 7A, B**). However this treatment led to an increased killing of these microbes. *M. smegmatis* was found to be killed significantly even at the 4 h time-point in Hsp70 treated macrophages, relative to untreated macrophages (**Fig. 7A**). With *M. avium* an appreciable effect on killing was observed at the 24 h time-point (**Fig. 7B**). In a separate experiment, we tested *M. avium* -infected cells up to 3 days after infection. As shown in **Fig. 7C** in the absence of Hsp70, killing was observed until 24 h that was followed by a period of growth until day 3. Following Hsp70 addition, there was less bacterial survival at days 1 and 3 (**Fig. 7C**).

## Discussion

Exosomes have recently gained attention because of their critical role in anti-tumor therapy. These vesicular organelles are released from MVBs when the latter fuse with plasma membrane upon various physiological and pathological conditions, such as heat shock and stress. The importance of exosomes has also been emphasized in anti-bacterial strategies, where antigens carried by these exosomes can be used for vaccination against different infectious diseases [42]. Of interest to the current study were recent reports showing that exosomes derived from macrophages infected with intracellular pathogens, especially mycobacteria, are pro-inflammatory in nature [10], [15].

We observed that exosomes that were derived from *M. smegmatis* or *M. avium* -infected macrophages (Fig. 1A, B) when added to uninfected macrophages stimulated the translocation of the NF-κB subunit p65 into the nucleus, a reflection of an increased inflammatory response. This was also evident in the increased synthesis and release of TNFα to the supernatant, relative to exosomes derived from control-uninfected cells (**Fig. 1C, D**). One rationale for the pro-inflammatory nature of exosomes derived from mycobacteria - infected cells is due to their endocytic origin; it is known that there is constant flow of membrane components between endosomes and phagosomes. During this flow, bacterial cell wall components are shed off during their residence in phagosomes, and traffic to endosomes [8], [9]; from MVBs they can conceivably reach exosomes that can be released to the extracellular media. These released exosomes (carrying bacterial components) can then potentially interact with uninfected cells to activate different signaling pathways [10]. Here, we could not detect mycobacterial LAM in exosomes from *M. smegmatis*- infected cells and only trace amounts in *M. avium*- infected ones using an antibody that recognizes both Ara-LAM and Man-LAM. This argues that other exosome components were responsible for the pro-inflammatory behavior of the exosomes from mycobacterial-infected cells.

Indeed, a prominent macrophage molecule that was easily detectable on exosomes by Western blotting and by immuno-EM was Hsp70, a member of the heat-shock family of proteins (Hsp family). Although Hsp70 acts mainly as an intracellular cytoplasmic chaperone responsible for facilitating folding of newly synthesized or mis-folded proteins, a fraction can be presumably packaged into MVBs that form exosomes. Hsp70 is also known to be released from tissues during necrosis so both the free molecule and the exosome-associated one appear to be physiologically important in the inflammatory, and other responses [43]. We observed that three-to-four times more exosomes are released from infected macrophages as compared to control-uninfected macrophages (**Fig. 1B** **and** **Table 1**). Also, infected macrophages released twice more Hsp70 in exosomes than that released in exosomes from control macrophages (**Fig. 2A**).

Our EM data showed that a fraction of this exosome Hsp70 could be detected on the outer surface of these vesicles, a site where they would be well-suited to interact with cell surfaces and play an immuno-modulatory role (**Fig. 2B**). In support of this notion exposure of exosomes to trypsin led to a significant reduction in Hsp70 levels in immunoblots (**Fig. 2C**). Indirect immunolabeling also showed a marginal increase in total Hsp70 levels after infection with either *M. smegmatis* or *M. avium* (**Fig. 2D,E**). We also observed by Western blotting of total cell lysates from control and mycobacterial infection conditions that the protein is significantly up-regulated at 24 h of infection compared to control uninfected cells (**Fig. 3A, B**). These data argue that in response to mycobacterial infection the total cellular Hsp70 is up-regulated and a fraction of this pool is released by the cells via exosomes. Interestingly, this was independent of whether bacteria were live or dead. However, treatment with mycobacterial lipids such as Ara-LAM or Man-LAM or total mycobacterial-lipids did not drastically induce Hsp70 expression at 24 h (**Fig. 3C, D**). These data also argue that any mycobacterial-lipids that may have been shed-off within macrophage phagosomes are unlikely to influence Hsp70 levels. LPS, a strong pro-inflammatory mediator, significantly increased Hsp70 levels at both 1 h and 24 h (**Fig. 3E, F**).

Many studies have identified an important immunological role for Hsp70 outside cells. Hsp70 is able to modulate the immune system by binding to different cell surface receptors, including Toll-like receptors (TLRs) that are known to activate NF-κB [33], [36], [37], and CD40 leading to the activation of p38 MAP kinase [44]. Similarly mycobacterial Hsp70 has been shown to signal through CCR5 chemokine receptor leading to the release of pro-inflammatory mediators from dendritic cells [45]. We found that treatment with Hsp70 led to some NF-κB activation in uninfected cells but the activation was significantly enhanced in mycobacteria-infected cells (**Fig. 4B, C**). Similarly, Hsp70 treatment of infected macrophages led to a significant increase in the release of TNFα (**Fig. 4D, E**). Other studies have also described significant NF-κB activation by Hsp70 in THP-1 monocytes at 100 ng/ml, which is similar to the concentration used in our experiments [24]. Our results are also in agreement with another study that showed enhanced TNFα release in human monocytes-derived-macrophages exposed to recombinant Hsp60 or Hsp70 [46].

Phagocytosis can be considered to be part-of the pro-inflammatory response and exosomes have been shown to stimulate this process [10], [15]. We found that whereas the co-incubation of exogenous Hsp70 with latex beads did not affect the rate of phagocytosis or their maturation (**Fig. 5A, D**), macrophages pre-incubated with Hsp70 for 2 h before addition of beads showed a higher rate of phagocytosis (**Fig. 5B**). Earlier studies have also provided indirect evidence that the extracellular presence of Hsp70 can induce increase in phagocytosis of opsonized bacteria [27], [28]. Another study also reported similar increase in phagocytosis of yeast particles after addition of purified mouse Hsp70 to cells. Competitive experiments performed by co-incubating Hsp70 with several known TLR7 ligands led to decrease in the rate of phagocytosis indicating Hsp70-TLR7 interaction on plasma membrane [28]. In addition to increasing the rate of phagocytosis our data show conclusively that pre-treatment of RAW264.7 macrophages with Hsp70 also leads to an increased maturation of the latex-bead phagosomes, as seen by an increased acquisition by phagosomes of the late endosome-lysosome marker LAMP2 (**Fig. 5C, E**). Thus, the cascade of signaling events induced by Hsp70 interaction with cell surface receptors include those that control the complex series of events that lead to phago-lysosome fusion.

When we investigated the effect of pre-incubation of macrophages with Hsp70 before infection with *M. smegmatis* and *M. avium* we saw no effect of the chaperone on the rate of uptake of either bacteria. However, as with latex beads we saw a significant increase in the rate of acquisition of LAMP2 by the mycobacterial phagosomes under this condition (**Fig. 6A–D**). Similarly, there was also a significant increase in DQ-BSA-positive mycobacterial phagosomes, indicating that these phagosomes were proteolytically active and presumably fused with lysosomes, in macrophages treated with Hsp70 (**Fig. 6E–H**). In many studies, as the fraction of matured phagosomes containing mycobacteria increases, there is a tendency for more bacteria to be killed by the exposure to the lysosome-like environment [31], [39], [40], [47]. In agreement with this observation, we saw that pre-treatment of macrophages with Hsp70 before infection with *M. smegmatis* led to more killing at the 4 h infection period (**Fig. 7A**), a time when macrophages are highly bactericidal towards *M. smegmatis* [40]. With the pathogen *M. avium*, the growth dynamics were different; in untreated macrophages there is a tendency for the bacteria to be killed until day 1 after infection, after which the bacteria begin to grow [41]. At both 1 and 3 days after infection there were significantly fewer surviving bacteria after Hsp70 pre-treatment (**Fig. 7B, C**).

In summary our study shows that infection with mycobacteria leads to an up-regulation of Hsp70 and to a significant release of this molecule to the extracellular medium via exosomes. Our data confirm that Hsp70 has pro-inflammatory properties in that it can stimulate NF-κB activation and the synthesis and release of TNFα. Pre-incubation of macrophages with Hsp70 stimulates the phagocytic uptake rate of latex beads, but not of mycobacteria. However, this treatment stimulates the maturation of phagosomes containing latex beads, *M. smegmatis* and *M. avium*. In the case of mycobacteria an important consequence of the enhancement of phagosome maturation is that it correlates with an increase in the rate of bacterial killing. Overall our data fit into an emerging picture revealing that exosomes, and their major component Hsp70 are important activators of many functions related to the pro-inflammatory response.

## Materials and Methods

### Reagents

All reagents were obtained from Sigma unless otherwise stated. The following antibodies were used: anti-p65 (Santa-Cruz, Heidelberg, Germany), anti-Hsp70 (BD Biosciences & Assay Designs, Ann Arbor, MI, USA), anti-LAMP2 (Iowa hybridoma bank, Iowa city, IA). The following secondary antibodies were used: anti-mouse–Alexa-Fluor 488 (Molecular Probes, Eugene, OR), anti-rat cy3 and anti-mouse cy3 (Jackson research laboratories, USA) and HRP-conjugated antibodies (Sigma). DQ-BSA was a generous gift from Francesca Peri (EMBL, Heidelberg). Mycobacterial cell wall lipids and anti-LAM antibodies were obtained as part of NIH-NIAID contract NO1-AI-40091. Recombinant mouse Hsp70 (Cat # ESP-502F) and bovine Hsp70 (Cat # H9776) were obtained from Assay designs and Sigma, respectively. Latex-beads (3 µm) were obtained from Polysciences, USA.

### Mycobacteria and cell culture

RAW264.7 mouse macrophages were cultured as described previously [40]. *M. smegmatis* mc^2^155 harboring a p19- (long-lived) EGFP plasmid and *M. avium* MAC 101 were grown as previously described [41], [47]. Briefly, *M. smegmatis* was grown in Middlebrook's 7H9 broth medium (Difco, Detroit, USA) supplemented with 0.5% glucose and 0.05% Tween-80 at 37°C on a shaker at 200 r.p.m. *M. avium* was grown in 7H9 broth additionally supplemented with 10% OADC (v/v) until exponential phase at 37°C/5% CO_2_ incubator. Media were supplemented with 50 µg/ml hygromycin (Roche, Germany) for selection of recombinant mycobacteria.

### Mycobacterial infection of macrophages

Bacterial cultures in exponential phase were pelleted, washed twice in PBS and re-suspended in DMEM media. Clumps of bacteria were removed by mild-ultrasonication for 10 min followed by a low-speed centrifuge for 1 min. Confluent macrophage cultures (2×10^8^ for exosome-isolation and 5×10^5^ for CFU assays) were infected with single-cell suspension of mycobacteria at multiplicity of infection (MOI) of 10∶1 (OD_600_ of 0.1). Under these conditions, >70% of the cells were infected with an average of 1–3 bacilli/cell. After 1 h or 3 h of uptake, cells were washed with PBS and replaced with DMEM containing 10 µg/ml of gentamicin to kill extracellular bacteria. After different times, cells were lysed with sterile water. Serial dilutions of lysates were plated on 7H10 agar plates and colonies were counted after 3 days (*M. smegmatis*) or 7 days (*M. avium*). Cells grown on coverslips and infected as above with GFP - *M. smegmatis* or rhodamine-labeled–*M. avium* were fixed at different times and immuno-labeled with anti-LAMP2 antibody or pre-incubated with 10 µg/ml of DQ-BSA 2 h before infection. For exosome preparations from *M. smegmatis* or *M. avium* - infected cells, macrophages were grown and infected in cell culture flasks and after 48 h supernatants were collected and processed further as described below.

### Exosome isolation

Exosomes were isolated from control, *M. smegmatis* and *M. avium* infected or LPS-treated (100 ng) or latex-bead fed cells using standard procedure [10], [48]. Briefly, cells were cultured in DMEM media that was centrifuged at 100,000 g for 15 h to deprive it of endogenous exosomes present in fetal calf serum. After 48 h of infection the supernatant was collected and subjected to differential centrifugation steps of 1200 g for 10 min, 3000 g for 10 min, 10,000 g for 30 min and finally 100,000 g for 2 h. The pellet so obtained was floated on a sucrose gradient that was centrifuged for 100,000 g for 2 h followed by a PBS wash. The amount of exosomes in each sample was measured by Micro-BCA kit from Pierce (Rockford, IL, USA).

### Electron microscopy

A drop of each exosome preparation (control, *M. smegmatis* and *M. avium*) was placed on parafilm and directly covered by a 300 mesh copper grid. After 10 min of incubation/attachment of the exosome sample to the formvar-coated grid, a quick washing was performed with PBS followed by 15 min incubation in blocking buffer (0.8% BSA and 0.1% fish skin gelatin in PBS). The grids were incubated with primary antibody against HSP70 (1∶10; anti-rabbit) and 10 nm protein A-gold for 30 min. To try to fix the gold labeling, 2% glutaraldehyde was used for 5 min followed by washing with double distilled water for 10 min. The grids were stained/embedded with a mixture of methylcellulose and uranyl acetate and analyzed by transmission EM. Quantitation of Hsp70 labeling on exosomes was done by manually counting the number of gold label/vesicles on 20 systematically selected images per sample.

### Immunoblotting

Whole-cell extracts were prepared at different times after *M. smegmatis* or *M. avium* infection (using either live or heat-killed bacteria) or from latex-bead incubated cells, by washing the cells in PBS followed by incubation in RIPA-lysis buffer for 30 min on ice. Similarly, protein lysates were also prepared from cells that were exposed to Ara-LAM (1 and 10 µg/ml), Man-LAM (1 and 10 µg/ml), total mycobacterial-lipids (20 and 50 µg/ml) and LPS (100 ng/ml). The lysates were centrifuged at 10,000 g for 15 min to pellet cell debris. After addition of 2x Laemlli buffer, equal concentration of protein was resolved on 10% SDS-PAGE followed by immunoblotting with anti-Hsp70 and anti-actin antibodies on nitrocellulose membrane. The bands were visualized using enhanced chemiluminescence (Roche, Germany). Equal volume of exosome preps from control, *M. smegmatis* and *M. avium* infected macrophages were resolved on 10% SDS-PAGE and either stained with SilverQuest silver-staining kit (Invitrogen, Carlsbad, CA, USA) according to manufacturer's instructions or immunoblotted with anti-Hsp70 antibody (Assay Designs, Ann Arbor, MI, USA). Densitometric analysis of the bands was performed using Image J program.

### Addition of exosomes to macrophages

Concentration of exosomes in different preparations was measured by Micro-BCA kit (Pierce, Rockford, IL, USA). 10 µg of each exosome was incubated with control cells. After 24 h, cells were fixed and immuno-labeled with anti-p65 antibody and supernatants were assayed for TNFα quantity using ELISA.

### Trypsin sensitivity assay

The surface presence of Hsp70 was confirmed by exposure of exosomes to 0.25% trypsin for 5 min at 37°C. After 5 min, equal volume of growth medium (exosome-free) was added to neutralize the trypsin activity [49]. Cells were then washed in PBS at 100,000 g for 2 h to recover the exosomes.

### Indirect immunofluorescence

Cells were fixed in 3.7% paraformaldehyde in PBS for 20 min and quenched by incubating with 50 mM NH_4_Cl in PBS. Subsequently, the cells on coverslips were blocked and permeabilized simultaneously in 0.1% saponin and 1% BSA in PBS. Washings with PBS were performed after each step and primary and secondary antibody incubations were done each for 1 h at room temperature. Cells on coverslips were mounted on slides with Dako mounting medium and analyzed with Zeiss LSM510 and Leica SP2 AOBS confocal microscope.

### ELISA

Supernatants collected from different experimental conditions were centrifuged at 2000 g for 10 min to get rid of cells. The supernatants were then stored at −80 degrees until assayed for TNFα (BD biosciences) and IL-1β (Pierce, Rockford, IL, USA) according to manufacturer's instructions.

### Phagocytosis assay with latex beads

RAW 264.7 macrophages were plated on coverslips one day before experiment. Cells, incubated or not with Hsp70, were fed with 3 µm IgG-coated latex-beads. After different times of bead uptake, cells were washed with PBS and fixed with 3.7% paraformaldehyde in PBS. Subsequently, the coverslips were blocked with 1% BSA in PBS and outside beads were stained with anti-IgG-AF488. After permeabilization with 0.1% saponin in PBS, the cells were labeled with anti-LAMP2 antibody. Total number of beads, beads that are inside the cells and LAMP2–positive latex-bead phagosomes were counted from at least 20 fields per coverslip on an Axiovert 200 fluorescent microscope. The results represent mean ± SEM of two independent experiments.

### Statistical analysis

Data are represented as mean ± SEM of three independent experiments. Asterisks indicate significant differences as determined by student's t-test. *P<0.05; **P<0.01.

## Supporting Information

Figure S1 Dot-blot of exosomes. Titration of purified Ara-LAM along with 10 µg of each kind of exosomes from control, M. smegmatis (MS), or M. avium (MAC) - infected cells was done on a PVDF membrane using dot-blot apparatus. The membrane was then subjected to incubation with anti-LAM and anti-actin antibody.
(0.08 MB TIF)

Figure S2 Exosome isolation from LPS treated and latex-bead incubated cells. RAW 264.7 cells were either fed latex-beads or incubated with LPS (100 ng/ml). After 48 h, exosomes were isolated and subjected to SDS-PAGE electrophoresis followed by incubation with anti-Hsp70 or anti-actin antibody.
(0.04 MB TIF)

Figure S3 Measurement of IL-1β by ELISA. RAW 264.7 cells were primed with LPS for 2 h followed by treatment with Hsp70. After 24 h, supernatants were assayed for IL-1β. Data represents mean ± SEM from three independent experiments.
(0.15 MB TIF)
