# Supplementary figures and images for: Exosomal Hsp70 Induces a Pro-Inflammatory Response to Foreign Particles Including Mycobacteria
Source: PLoS One. 2010 Apr 12;5(4):e10136. doi: 10.1371/journal.pone.0010136 (PMC2853569; doi:10.1371/journal.pone.0010136)

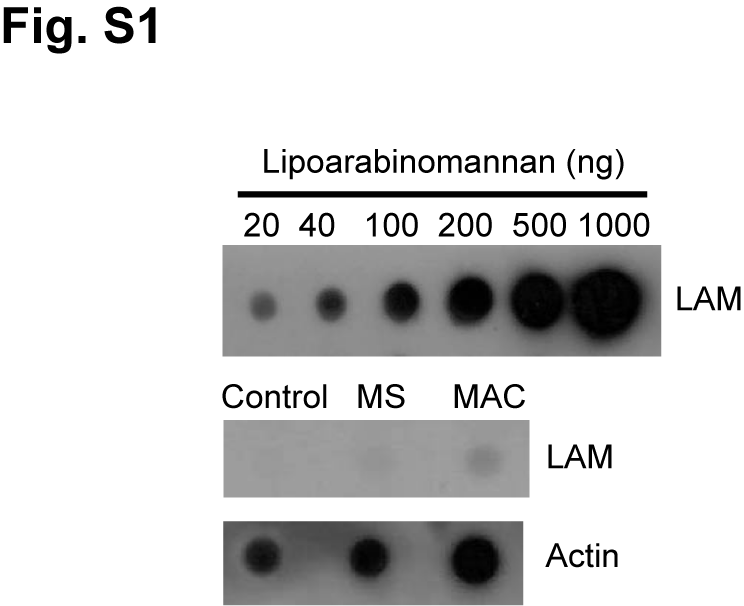

Supplement: Figure S1 — Dot-blot of exosomes. Titration of purified Ara-LAM along with 10 µg of each kind of exosomes from control, M. smegmatis (MS), or M. avium (MAC) - infected cells was done on a PVDF membrane using dot-blot apparatus. The membrane was then subjected to incubation with anti-LAM and anti-actin antibody. (0.08 MB TIF) [file pone.0010136.s001.tif]

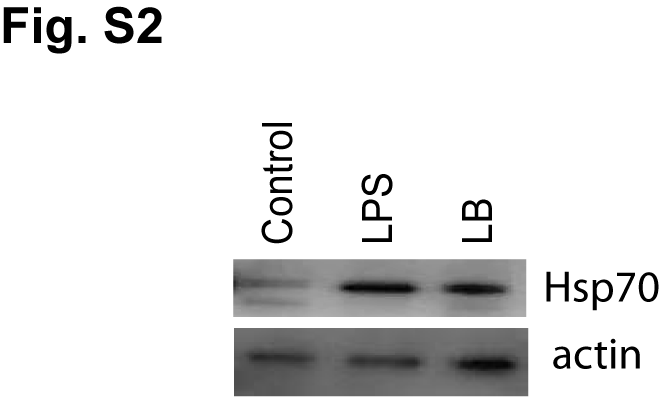

Supplement: Figure S2 — Exosome isolation from LPS treated and latex-bead incubated cells. RAW 264.7 cells were either fed latex-beads or incubated with LPS (100 ng/ml). After 48 h, exosomes were isolated and subjected to SDS-PAGE electrophoresis followed by incubation with anti-Hsp70 or anti-actin antibody. (0.04 MB TIF) [file pone.0010136.s002.tif]

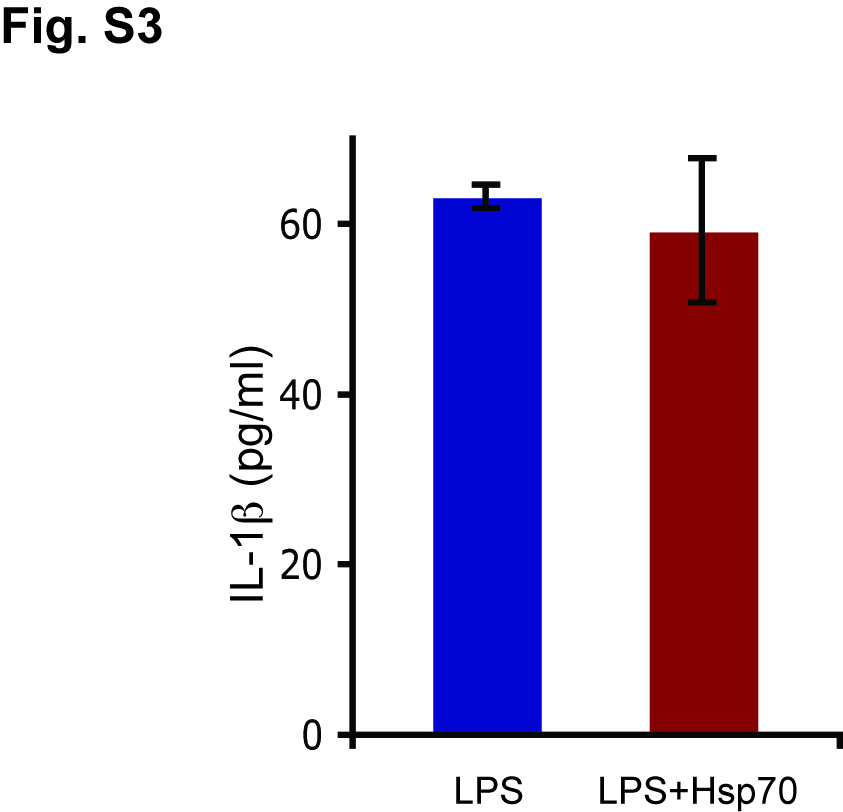

Supplement: Figure S3 — Measurement of IL-1β by ELISA. RAW 264.7 cells were primed with LPS for 2 h followed by treatment with Hsp70. After 24 h, supernatants were assayed for IL-1β. Data represents mean ± SEM from three independent experiments. (0.15 MB TIF) [file pone.0010136.s003.tif]
